# Evaluation of Antibacterial Activity of Selenium Nanoparticles against Food-Borne Pathogens

**DOI:** 10.3390/microorganisms11061519

**Published:** 2023-06-07

**Authors:** Qunying Yuan, Rong Xiao, Mojetoluwa Afolabi, Manjula Bomma, Zhigang Xiao

**Affiliations:** 1 Department of Biological and Environmental Science, Alabama A&M University, Huntsville, AL 35762, USA; rong.xiao@aamu.edu (R.X.); mojetoluwa.afola@bulldogs.aamu.edu (M.A.); manjula.bomma@aamu.edu (M.B.); 2 Department of Electrical Engineering and Computer Science, Alabama A&M University, Huntsville, AL 35762, USA; zhigang.xiao@aamu.edu

**Keywords:** selenium, nanoparticles, antibacterial, food-borne pathogen

## Abstract

Selenium is an essential micronutrient for all mammals and plays an important role in maintaining human physiological functions. Selenium nanoparticles (SeNPs) have been shown to demonstrate antioxidant and antimicrobial activity. The objective of this study was to explore whether SeNPs have the potential to be used as food preservatives with which to reduce food spoilage. SeNPs were synthesized through ascorbic acid reduction of sodium selenite (Na_2_SeO_3_) in the presence of bovine serum albumin (BSA) as a capping and stabilizing agent. The chemically synthesized SeNPs had a spherical conformation with an average diameter of 22.8 ± 4.7 nm. FTIR analysis confirmed that the nanoparticles were covered with BSA. We further tested the antibacterial activity of these SeNPs against ten common food-borne bacteria. A colony-forming unit assay showed that SeNPs exhibited inhibition on the growth of *Listeria Monocytogens* (ATCC15313) and *Staphylococcus epidermidis* (ATCC 700583) starting at 0.5 µg/mL, but higher concentrations were required to slow down the growth of *Staphylococcus aureus* (ATCC12600), *Vibrio alginolyticus* (ATCC 33787), and *Salmonella enterica* (ATCC19585). No inhibition was observed on the growth of the other five test bacteria in our study. Our data suggested that the chemically synthesized SeNPs were able to inhibit the growth of some food-borne bacteria. The size and shape of SeNPs, method of synthesis, and combination of SeNPs with other food preservatives should be considered when SeNPs are to be used for the prevention of bacteria-mediated food spoilage.

## 1. Introduction

Microorganism-mediated food spoilage not only results in food waste, but also causes food-borne diseases. The application of food preservatives, along with strict preservation processes, is a common practice for suppressing the growth of microorganisms in order to increase food safety and reduce food waste. In recent years, SeNPs, which have shown both antioxidant and antimicrobial activities [1,2,3,4,5,6,7], gained attention for their potential applications toward protecting food quality and reducing food spoilage [8,9,10].

Selenium, an essential micronutrient for all mammals, is required for maintaining good human health due to its antioxidant activities. Selenium fulfills its physiological functions through its incorporation into selenoproteins [11,12,13]. The most characterized selenoproteins are glutathione peroxidases, selenoprotein P, thioredoxin reductase, and thyroxine 5-deiodinase [7,11,12,13]. Glutathione peroxidases, along with other enzymes such as catalase and superoxide dismutase, prevent oxidative damage to cells by eliminating reactive oxygen species; meanwhile, selenoprotein P is involved in the detoxification of heavy metals and free radicals, and is responsible for redistributing selenium in the brain [14,15]. Thioredoxin reductases regulate redox reactions by providing selenide for the synthesis of selenoproteins [7,16]. These enzymes are intracellular antioxidant molecules important in preventing cancer, but which are also involved in tumor development [17]. Thyroxine 5-deiodinase carries out the production of active thyroid hormone. A deficiency of selenium has been associated with increases in viral infection, cardiovascular disease, infertility, myodegenerative diseases, and cognitive decline [11,17,18], as well as thyroid autoimmune diseases [19]. Due to the important roles of selenium in human physiological functions, the potential applications of selenium in food supplements, cancer-preventatives, and drug delivery systems, as well as antimicrobial and anti-inflammatory agents, are being actively investigated in biomedicine and food sciences [2,3,5,7,9,10,17,20,21]. Nanosized selenium is of particular interest due to its better biocompatibility, higher bioavailability, lower toxicity, and other advantages [22,23,24,25]. SeNPs displayed antioxidant activity in various studies and have been tested for their utilization in the treatment of cancer and other diseases [2,3,20,26]. In addition, it has been reported that SeNPs were able to inhibit human pathogenic bacteria, including *B. cereus*, *MRSA*, *S. aureus*, *S. agalactaie*, *E. coli*, *E. faecalis*, and *L. monocytogenes* [4,8,10,27,28,29]. It is noteworthy that some of them, such as *E. coli* and *L. monocytogenes*, are common food-borne bacteria. If SeNPs are able to serve as food additives, they would not only reduce food deterioration by both hindering microbial growth and preventing food oxidation, but also benefit consumers with selenium deficiency. Thus, it is important to test whether SeNPs are able to inhibit the growth of a wider range of food-borne bacteria. This study aimed to examine whether SeNPs can inhibit the growth of some common food-borne bacteria and explore their potential in food preservation. 

In this study, we synthesized SeNPs using vitamin C to reduce SeO_3_^2+^, with BSA as stabilizing agent, and further tested the antibacterial effect of these SeNPs against a total of 10 types of bacteria: *Salmonella enterica* (ATCC19585), *Salmonella enterica* (ATCC49284), *Listeria Monocytogens* (ATCC15313), *Staphylococcus epidermidis* (ATCC 700583), *Staphylococcus aureus* (ATCC12600), *Vibrio alginolyticus* (ATCC 33787), *Enterococcus faecalis* (ATCC 19433), *Enterobacter cloacae* (ATCC3044), *Vibrio parahaemolyticus* (ATCC49529), and *Escherichia coli* (ATCC2326). Our results suggested that SeNPs inhibited the growth of *Listeria Monocytogens* (ATCC15313), *Staphylococcus epidermidis* (ATCC 700583), *Staphylococcus aureus* (ATCC12600), *Vibrio alginolyticus* (ATCC 33787), and *Salmonella enterica* (ATCC19585). This is the first study that tested the antibacterial effects of chemically synthesized SeNPs against such a wide range of food-borne bacteria. Our results suggested that the criteria for selecting SeNPs suitable for the development of food preservatives should include the size, morphology, method of synthesis, and surface chemical properties of SeNPs. Combining SeNPs with other food preservatives may be needed to prevent food spoilage.

## 2. Materials and Methods

### 2.1. Synthesis of SeNPs

SeNPs were synthesized following the procedure described by Chung [30], with modifications. Vitamin C was used to reduce Na_2_SeO_3_, and BSA was used to cap and stabilize the nanoparticles. Briefly, 18 mL of 50 mM of vitamin C was added dropwise to 2 mL of 100 mM Na_2_SeO_3_ containing 10 mg/mL BSA, with continuous shaking at 100 rpm for 30 min at room temperature. Subsequently, SeNPs were collected by centrifugation at 17,000× *g* for 1 h and further washed three times with pure water. The SeNPs were freeze-dried and stored at 4 °C for future use. In order to make the desired concentration of SeNPs, the dried nanoparticles were weighed and resuspended in an appropriate volume of pure water by sonication on ice. The resuspended SeNPs were stored at 4 °C and used within a month.

### 2.2. UV–Vis Spectroscopy

UV-Vis spectroscopy can be used to characterize the optical property of materials [31]. To help characterize the constitutes of the SeNPs using UV-Vis, SeNPs were washed and diluted with pure water. Thereafter, 2 mL aliquot of SeNPs was added in a 1 cm path length quartz cuvette and scanned using UV–Vis spectroscopy (Genesys 10 S UV-Vis spectrophotometer; Fisher Scientific, Pittsburgh, PA, USA) operated at a resolution of 1 nm in the range of 200–800 nm.

### 2.3. Fourier Transform Infrared (FTIR) Spectrometer Analysis of SeNPs

FTIR is a common method with which to characterize the surface chemical properties of nanoparticles [31]. FTIR was used to identify the possible chemical groups that cover the nanoparticles. A droplet of the concentrated SeNP solution in pure water was placed onto an infrared transmitting substrate and air dried to evaporate the solvent. The dried droplet was directly analyzed on a Thermo-Nicolet iS50 FTIR spectrometer equipped with a Continuum microscope in transmission mode. The analytical spot size was approximately 100 microns × 100 microns. OMNIC 7.1 software was used to perform data analysis. The FTIR spectrometer analysis of SeNPs was carried out by Eurofins EAG Materials Science, Sunnyvale, CA, USA.

### 2.4. Zeta Potential Measurements of SeNPs

Zeta potential measures the electrical potential at the solid–liquid interface [32], and it is often used to evaluate the stability of nanoparticles [2,4,6,10]. In this study, we measured zeta potential in order to estimate the stability and possible charges on the surfaces of the nanoparticles. The nanoparticles were washed with pure water and further dispersed in pure water for zeta potential analysis. Zeta potential was recorded using Malvern Zetasizer Nano ZS system at temperature of 25 °C (Particle Technology Labs, Downers Grove, IL, USA).

### 2.5. Scanning Transmission Electron Microscopic Characterization

STEM has great capabilities for the study of the size, morphology, and chemical composition of nanomaterials [31,33]. We used STEM to determine the morphological properties of the SeNPs. The samples for STEM analysis were resuspended in pure water. An FEI Tecnai F30 super-twin field-emission-gun transmission electron microscope (TEM) operating at 300 kV was used to obtain the TEM images, electron diffraction patterns, and annular dark-field (ADF) STEM images. Chemical composition was analyzed using an X-ray energy-dispersive spectrometer (EDS) attached to the TEM. A dual-beam FIB (FEI Helios 600 dual beam FIB) was used to obtain the SEM images of the SeNPs.

### 2.6. Antibacterial Activity of SeNPs

A colony-forming unit assay, with slight modifications, was employed to test the antibacterial activity of SeNPs. A single colony of bacteria was inoculated in 10 mL LB and cultivated at 37 °C with continuous shaking at 250 rpm for 18–20 h. Thereafter, 0.5 mL of the overnight culture was centrifuged and resuspended in 20 mL of fresh LB. Cells were grown at the same conditions until OD_600nm_ reached between 0.6 and 0.62. The bacterial cells were subsequently inoculated in fresh LB at a ratio of 1:20 in the presence of SeNPs at concentrations of 0, 0.5, 1, 2.5, 5, 10, 15 and 30 µg/mL, and further grown for 24 h, as described below. In general, the procedures were similar for each test bacterium, but conditions, such as growth temperature and serial dilution, were adjusted slightly for different bacteria so that the number of single colonies on each agar plate fell between 10 and 100. Bacteria were cultured for 24 h with continuous shake 250 rpm, at a temperature of 30 °C or 37 °C dependent on their growth rate. However, the conditions for growing the same bacterium were maintained the same throughout the study. Subsequently, serial 10× dilutions were made from the overnight bacterial culture: 100 µL of 1:1,000,000 or 1:10,000,000 dilution was plated on agar plates without antibiotics or SeNPs. Cells were grown at 37 °C for at least 16 h to obtain visible colonies. The plates containing colonies of between 10 and 100 bacteria were used to evaluate the antibacterial activity of SeNPs. Additionally, 100 µg/mL of kanamycin was generally used as a positive control, while 100 µg/mL of each carbenicillin and kanamycin were combined and used as a positive control for *Enterobacter cloacae* (ATCC3044).

### 2.7. Preparation of Bacteria for SEM Analysis

The initial preparation of bacteria was similar to that for the analysis of the antibacterial activity of SeNPs. The overnight bacterial culture was resuspended in fresh LB medium and grown to reach an OD_600nm_ around 0.6 for the subsequent cell preparation for SEM analysis. In the next step, 24-well plates were used for bacteria cultivation. A sterile 0.2 µm black polycarbonate membrane filter was placed at the bottom of a well, and an appropriate volume of fresh medium and SeNPs were added to the well to obtain a total volume of 950 µL and final concentrations of SeNPs at 5, 10, and 30 µg/mL, followed by the addition of 50 µL of bacteria. After incubation with SeNPs at 37 °C for 18–20 h with shaking at 250 rpm, the plates were allowed to sit at room temperature for 30 min. Thereafter, the membrane filters were removed and transferred to the wells of a new plate. The cells were processed for SEM analysis, following procedures with slight modifications [34]. Cells were fixed in 3% glutaraldehyde in 0.1 M phosphate buffer (pH 7.2) for 10 min. The cells were washed three times in 0.1 M phosphate buffer (pH 7.2), further postfixed in 1% osmium tetroxide (in H_2_O) for 1 h, and dehydrated once in 30%, 50%, 75%, and 90%, and twice in 100% ethanol, for 5 min each. The solution was removed using a multi-channel pipette after each step. The filters were mounted onto SEM stubs and sputter-coated with 10 nm gold for SEM examination.

A dual-beam FIB (FEI Helios 600 dual beam FIB) was used to obtain the SEM images of the bacterial cells, and cell size was analyzed using ImageJ 1.53a Software.

### 2.8. Statistical Methods

All data were expressed as mean ± SD. Comparison between groups was evaluated with Student’s *t* test. Probability values of <0.05 were considered significant.

## 3. Results

### 3.1. UV-Vis Spectrum of SeNPs

**Figure 1:**
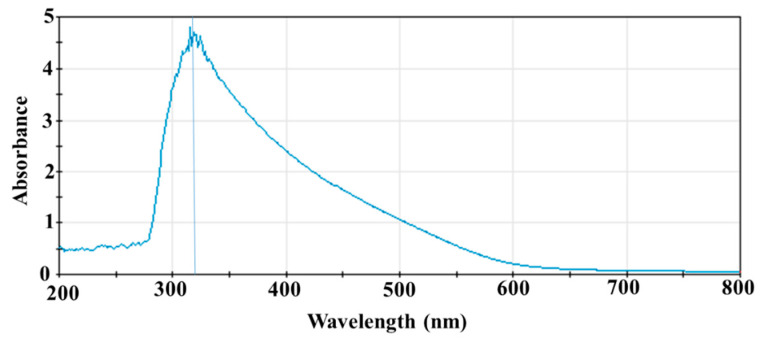
**UV-Vis Spectrum of SeNPs**. A typical spectrum of SeNPs that were washed with pure water and resuspended in pure water for UV-vis scans from 200 to 800 nm. Experiments were repeated three times.

An absorption peak was observed around 320 nm when SeNPs were scanned between 200 and 800 nm (Figure 1), which is consistent with the observed peak absorption of SeNPs synthesized using a green method [35]. It was also reported that the surface plasmon resonance (SPR) of SeNPs causes a maximum absorption between 200 nm and 400 nm in the optical absorption spectrum [36]; thus, the peak at 320 nm likely resulted from the SPR of SeNPs.

### 3.2. TEM and EDS Examination of SeNPs

**Figure 2:**
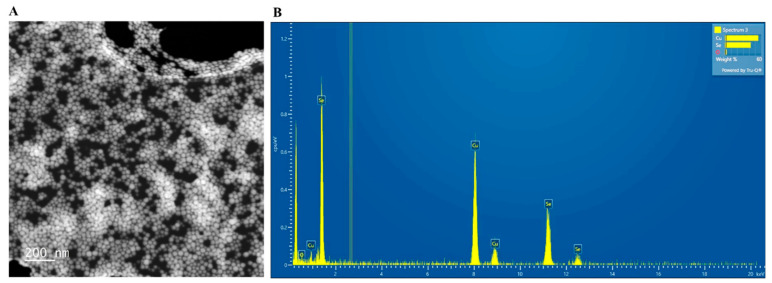
**TEM and EDS Characterization of SeNPs**. (**A**) TEM examination of SeNPs. SeNPs have a spherical shape and a diameter of 22.8 ± 4.7 nm. n = 207. (**B**) EDS analysis of SeNPs.

TEM analysis revealed that the conformation and dimension of the SeNPs were uniform, with a spherical shape and a diameter of 22.8 ± 4.7 nm (Figure 2A, n = 207 nanoparticles). Further EDS analysis identified that the nanoparticles were composed of selenium (Figure 2B). The spectrum also indicated that the sample may contain copper, which could be the background or contaminants from the support waffle.

### 3.3. FTIR of SeNPs

**Figure 3:**
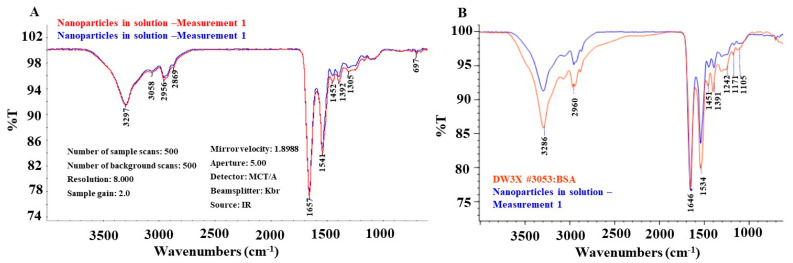
**FTIR Analysis of SeNPs**. (**A**) SeNPs resuspended in water were analyzed on a Thermo-Nicolet iS50 FTIR spectrometer equipped with a Continuum microscope in transmission mode. (**B**) The alignment of FTIR spectra between SeNPs and BSA, which was analyzed using the same system.

The dried SeNPs were analyzed by FTIR, and the spectra obtained from two different portions of the sample were provided in a spectrum overlay format in Figure 3A,B. The overlap between the spectra confirms the homogeneity of the sample (Figure 3A).

The sample was characterized as a biological polyamide, similar to bovine albumin, by comparison with references from the library of Eurofins EAG Materials Science (Sunnyvale, CA, USA). The recordings were also similar to what has been reported [37]. Specifically, the prominent peaks at ~3286, 2960, 1646, 1534, 1451, 1391, 1424, 1171, and 1105 cm^−1^ overlap with those of the bovine albumin reference (Spectrum in Figure 2B), supporting that they most likely have identical chemical properties. The identity of each peak is proposed as below and consistent with functional groups from proteins [37]. The peak at 3297 cm^−1^ was assigned to O-H or N-H stretch. The peak at 3058 cm^−1^ likely resulted from NH stretching vibrations of amide A. Peaks at 2956 cm^−1^ and 2869 cm^−1^ were assigned to -CH_2_-; symmetrical vibrations and -CH_2_- asymmetric vibrations, respectively. The peak at 1657 cm^−1^ was due to amide I, mainly its C=O stretching vibrations. The peak at 1541 cm^−1^ was from amide II, and its coupling of bending vibrations of N-H and stretching vibrations of C-N. The vibrational mode of N-H generated a peak at 1452 cm^−1^. Side chain COO^-^ could have a peak at 1392 cm^−1^. The peak at 1305 cm^−1^ resulted from the vibrational mode of C-N. C-N stretching/N-H bending vibrations resulted in a peak at 1241 cm^−1^. The peak at 1105 cm^−1^ was due to the vibration mode of C-O. The absorption bands observed in the region of 1000–800 cm^−1^ probably resulted from the C-H out-of-plane bending or wagging vibrations of hydrogen atoms attached to unsaturated carbons.

There were some shifts from the BSA’s typical absorption bands. The shifts were probably due to the conformation change in BSA caused by the interaction between BSA and SeNPs.

### 3.4. Zeta Potential Analysis

Zeta potential measurement can assess the surface charges of nanoparticles and estimate the degree of repulsion between the charged particles in colloid status. A zeta potential lower than −30 mV or higher than +30 mV usually suggests good colloid stability of nanoparticles due to the electrostatic repulsion between individual particles [38]. In addition, the charge status also has a significant impact on the affinity of SeNPs to bacterial cell membranes, and their subsequent entry into the bacteria. Zeta potential was determined using Malvern Zetasizer Nano ZS system (Particle Technology Labs, Downers Grove, IL, USA).

**Figure 4:**
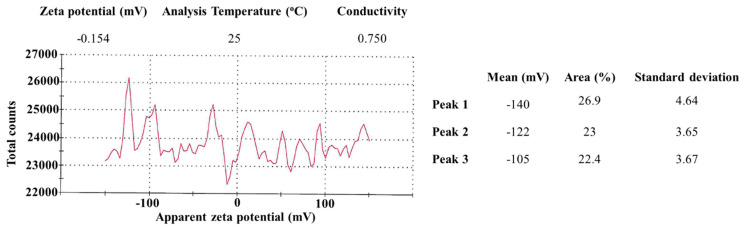
**Zeta Potential Measurement of SeNPs**. SeNPs were resuspended in pure water, and zeta potential was recorded using a Malvern Zetasizer Nano ZS system at a temperature of 25 °C. Results were summarized in the insets.

When the nanoparticles were dispersed in pure water at 25 °C, no main peaks were recorded (Figure 4). It appeared that there were three peaks at 140 mV, 122 mV, and 105 mV; however, the variance was quite large, and the count rate was low for each peak (Figure 4). The overall zeta potential of the SeNPs was −0.154 mV (Figure 4). Based on the high potential of selenium and BSA to dissolve in water, the low signals and near-zero zeta potential observed in this study indicated that the recordings were likely related to the carrier, water. The results can be extrapolated as indicating that SeNPs may be so soluble in water that the Zetasizer Nano ZS system cannot observe particles freely moving in Brownian motion, and, thus, cannot measure the zeta potential of SeNPs. On the other hand, the Zeta potential analysis may suggest that SeNPs might be quite stable due to their high solubility in water. It is interesting that the zeta potential result was different from the reported positive or negative potential of BSA-covered SeNPs [4,39] synthesized by the same method, which was measured when SeNPs were suspended in reaction buffer instead of water.

### 3.5. Antibacterial Activity of SeNPs

We tested the antibacterial activity of SeNPs against a variety of food-borne pathogens, including *Listeria moncytogens* (ATCC15313), *Staphylococus Aureus* (ATCC12600), *Staphylococus epidermidis* (ATCC 700583), *Enterococcus faecalis* (ATCC 19433), *Vibrio alginolyticus* (ATCC 33787), *Salmonella enterica* (ATCC19585), *Vibrio parahaemolyticus* (ATCC43996), *Salmonella enterica* (ATCC 49284), *Enterobacter cloacae* (ATCC BAA3044), and *Escherichia coli* (ATCC BAA2326). Our results are summarized in Table 1, as well as in Figures S1 and S2. Kanamycin was used as a positive control. Nine of the ten test bacteria were killed by kanamycin at 100 µg/mL, with the exception that *Enterobacter cloacae* (ATCC3044) survived both kanamycin and carbenicillin at a concentration of 100 µg/mL, suggesting that this strain developed multi-drug resistance.

SeNPs displayed a negative effect on the growth of three of the four Gram-positive test bacteria in our study (Table 1). SeNPs showed a 16% inhibition at 0.5 µg/mL against *Listeria moncytogens* (ATCC15313) (Table 1 and Figure S1). When the concentration reached to 1 µg/mL and above, there was a significant reduction in the number of *Listeria moncytogens* (ATCC15313) colonies (*p* < 0.0001 for all the remaining SeNP concentrations). SeNPs at 1 and 2.5 µg/mL reduced *Listeria moncytogens* (ATCC15313) colonies by 50% and 78%, respectively. The inhibitory effect maintained between 81 and 87% at concentrations from 10 to 30 µg/mL. SeNPs did not exhibit significant inhibition on the growth of *Staphylococus aureus* (ATCC12600) until its concentration reached 10 µg/mL. The colony-forming unit was reduced by 42.0% at 10 µg/mL (*p* < 0.05), 51.1% at 15 µg/mL (*p* < 0.01), and 59.4% at 30 µg/mL (*p* < 0.001) (Table 1 and Figure S2). SeNPs also displayed an adverse effect on the growth of *Staphylococus epidermidis* (ATCC 700583), with a 27.5% reduction at 0.5 µg/mL (*p* = 0.058) and a 37.5% reduction at 1 µg/mL (Table 1).

The inhibition on *Staphylococus epidermidis* (ATCC 700583) reached 58.1% at 5 µg/mL of SeNPs (*p* < 0.01 at all concentrations ≥ 1 µg/mL) (Table 1). However, the effect of SeNPs maintained at a similar level at concentrations from 10 to 30 µg/mL. It is surprising that SeNPs failed to hinder the growth of a Gram-positive bacterium, *Enterococcus faecalis* (ATCC 19433). SeNPs also exhibited some inhibitory effect against two Gram-negative bacteria, *Vibrio alginolyticus* (ATCC 33787) and *Salmonella enterica* (ATCC19585). For *Vibrio alginolyticus* (ATCC 33787), there was an 11% reduction in colony-forming units at 0.5 µg/mL, but a 43.8% decrease at 2.5 µg/mL was observed (*p* < 0.05). The inhibition on the growth of *Vibrio alginolyticus* (ATCC 33787) was maintained around 50% at all higher concentrations from 10 to 30 µg/mL (*p* ≤ 0.01). The effect against *Salmonella enterica* (ATCC19585) was even milder. The SeNPs only diminished the number of colonies of *Salmonella enterica* (ATCC19585) by about 30% at 15–30 µg/mL (*p* < 0.05). On the other hand, the SeNPs demonstrated no effects against other Gram-negative bacteria, including *Salmonella enterica* (ATCC49284), *Vibrio parahaemolyticus* (ATCC43996), *Enterobacter cloacae* (ATCC BAA3044), and *Escherica coli* (ATCC BAA2326) at any tested concentrations.

### 3.6. SEM Analysis of Bacteria Treated with SeNPs

SEM was used to examine the bacterial morphological structures after SeNP treatment to reveal the possible damages caused by SeNPs. We selected *Listeria monocytogens* (ATCC15313) to represent the Gram-positive bacteria, based on our observation that SeNPs showed greater inhibition in the growth of *Listeria monocytogens* (ATCC15313) than that of others, while we chose *Enterobacter cloacea* (ATCC BAA3044) as a representative of Gram-negative bacteria, since SeNPs did not exhibit a good antibacterial effect on this bacterium, and it also showed resistance to both kanamycin and carbenicillin in our experiments.

**Figure 5:**
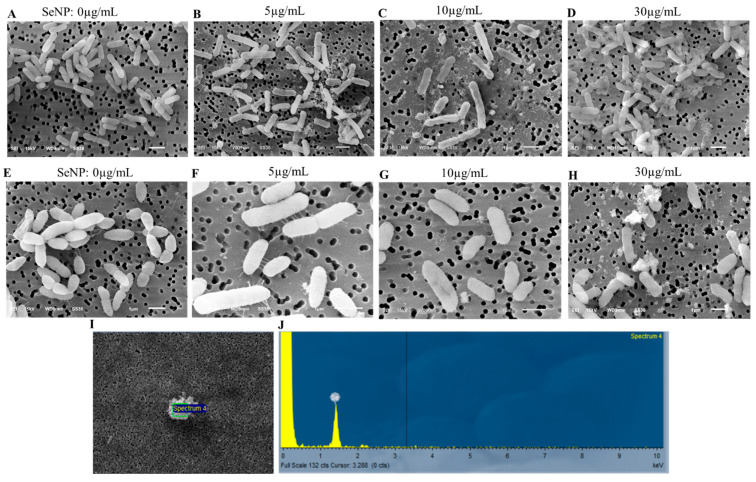
**SEM and EDS Characterization of Bacteria Treated with SeNPs** (**A**–**D**). Representative images of *Listeria moncytogens* treated with different doses of SeNPs. (**E**–**H**). Representative images of *Enterobacter cloacae* treated with different doses of SeNPs. (**I**) A typical clump observed under SEM was further analyzed by EDS. (**J**) EDS spectrum of the clump in (**I**). Experiments and SEM examination were repeated three times.

We observed morphological changes in both bacteria after SeNP treatment. Nevertheless, it appeared that a higher concentration of SeNPs was required in order to deploy similar damage to *Enterobacter cloacae* (ATCC BAA3044). As presented in Figure 5, cells of both *Listeria monocytogens* (ATCC15313) and *Enterobacter cloacae* (ATCC BAA3044), in the absence of SeNPs, showed normal rod-like shapes, smooth surfaces, and even filamentary structures around cells. However, SEM analysis detected more severe cell damage in *Listeria monocytogens* (ATCC15313) at each of the subsequently higher test concentrations (5, 10, and 30 µg/mL) (Figure 5). Many cells were dented, shrunken, and even disrupted (Figure 5B–D,G,H). Cell debris formed clumps to which SeNPs were attached (Figure 5D,E,H–J). *Listeria moncytogens* (ATCC15313) cell width, analyzed by ImageJ, did not show a significant change (Table 2). However, 30 µg/mL SeNP treatment appeared to significantly reduce the cell width of *Enterobacter cloacea* (ATCC BAA3044), compared to the untreated control (Table 2: SeNPs at 0 µg/mL: 640.8 ± 47.6 nm; SeNPs at 30 µg/mL: 594.9 ± 73.3 nm, *p* < 0.001). Moreover, EDS analysis detected high selenium content in the clumps of cell debris (Figure 5I,J).

## 4. Discussion

The applications of SeNPs as therapeutic agents in biomedicine have gained increasing interest. SeNPs displayed antioxidant, anticancer, antidiabetic, and antimicrobial activities in various studies [2,3,4,6,8,10,20,22,24,25,26,35,36]. The biofunctionality of SeNPs is determined by their morphology, size, surface chemical composition, and charges [4,6,40]. Therefore, the methods of SeNP synthesis, which control the physical and chemical properties of the SeNPs, are critical for the functionalization of SeNPs. SeNPs can be synthesized using physical, chemical, and biogenic methods [4,9,21,40]. Physical methods involve using laser radiation or ultrasound, which require specific equipment [21,40]. Biogenic methods take advantage of the reduction of organic molecules in extracts from plants or microorganisms to reduce selenium compounds in order to form SeNPs [2,9,10,21,26,27,28,41]. However, the extraction of plant chemical ingredients adds an extra step to the procedure, and a large reaction volume is usually needed when plant extracts are used [2,28,42]. While microorganism-mediated SeNP synthesis provides good yield, the separation of SeNPs from cellular debris is almost impossible, which will interfere with the end applications of SeNPs [41]. In this study, we chose to use vitamin C to reduce Na_2_SeO_3_, and BSA as a capping and stabilizing agent, to produce SeNPs for three main reasons. Firstly, it is a simple, fast, and productive method without the need for specific equipment [4,6,30,43]. Secondly, the size of the SeNPs can be controlled by adjusting the molar ratio of the two reactants, vitamin C and Na_2_SeO_3_, and the speed at which the reaction is stirred [30]. Lastly, the SeNPs are coated with a soluble biological molecule, BSA, which increases their solubility, stability, activity, and biocompatibility [4,6,30]. UV-vis spectroscopy (Figure 1) and STEM (Figure 2A,B) verified that we successfully produced SeNPs using this method. The resulting spherical SeNPs had an average diameter of 22.8 ± 4.7 nm (Figure 2), which should allow them to interact with bacterial surfaces and penetrate cell membranes [40,44,45]. In addition, the results of FTIR supported that the SeNP surfaces were covered by BSA (Figure 3).

SeNPs have been shown to exhibit antibacterial activity against a wide range of microorganisms, including bacteria, viruses, and fungi [2,4,8,10,27,28,43,46]; however, our study is the first one that examined the antibacterial effect of SeNPs synthesized by vitamin C reduction of Na_2_SeO_3_ against as many as 10 common food-borne bacteria, and also utilized SEM to help elucidate the possible mechanisms of the antibacterial action of SeNPs. Among the 10 test bacteria selected for the present study, the SeNPs showed the strongest effect against *Listeria monocytogens* (ATCC15313), with a 50% inhibition at 1 µg /mL, which reached ~80% inhibition at 10 µg/mL. SeNPs even inhibited the growth of *Staphylococcus aureus* (ATCC12600) and *Staphylococcus epidermidis* (ATCC 700583) by 50%, albeit at a higher concentration. Unfortunately, they did not display notable effects against the Gram-negative bacteria, with the exception of mild inhibition of *Vibrio alginolyticus* (ATCC 33787) and *Salmonella enterica* (ATCC19585) (Table 1).

It has been thought that SeNPs deployed antibacterial activity by three main mechanisms: (1) impairing cell walls and membranes; (2) penetrating through cell membranes and subsequent interference in the activities of biological molecules; and (3) increasing oxidative stress [40]. All three mechanisms may contribute to the antibacterial activity of SeNPs in the present study. Initially, SeNPs have to be able to adhere to bacteria in order to act on them. This first-step interaction between SeNPs and bacteria depends on the charges and molecular components of both SeNPs and bacterial cell walls/cell membranes [40]. The Gram-negative bacteria have an outer cell membrane composed of lipopolysaccharides, and an inner cell wall that has a thin layer of peptidoglycans, while the cell wall of Gram-positive bacteria is usually thicker and mainly consists of peptidoglycans [47,48]. The cell walls of both Gram-positive and Gram-negative bacteria are negatively charged; however, Gram-negative bacterial cell walls in general are more negatively charged [48,49,50].

We hypothesized that zeta potential examination would provide information regarding the charge status of the BSA-covered SeNPs. However, zeta potential analysis indicated that the average potential of BSA-covered SeNPs was −0.154 mV (Figure 4), due to the great variation and low signals during the zeta potential measurement. The near-0 mV zeta potential of SeNPs was surprising, since the SeNPs were covered by BSA (Figure 4), which is supposed to be negatively charged in pure water that has a pH of about 7. We speculated that the high solubility of both BSA and selenium in water likely granted the BSA-coated SeNPs high solubility in water, and the interface between the carrier (water) and nanoparticles may not have been easily formed; thus, it was not easy for the Zetasizer Nano ZS system to detect the nanoparticles freely moving in Brownian motion and measure their zeta potential. While zeta potential could not provide evidence to support a strong interaction between SeNPs and bacterial cell walls or cell membranes, albumin itself was demonstrated to be able to bind to the peptidoglycans, lipopolysaccharides, and lipoteichoic acid of the lipid bilayer of mammalian cells with high affinity [51]. In addition, the interaction between BSA and cell walls may change the conformation of both BSA and the biomolecules on cell membranes [52], increasing the adhesion between bacterial cell walls and BSA-covered SeNPs. Even if the overall surface charge of SeNPs was slightly negative, they can interact with bacterial surfaces through weak regional interactions, such as hydrogen bonds, van der Waals interactions, electrostatic interactions, and hydrophobic interactions [53]. The higher peptidoglycan content in Gram-positive bacterial cell walls would possibly have a stronger interaction with the slightly negatively charged BSA-coated SeNPs, which may cause greater toxicity to the cell walls and higher cell membrane permeability, inducing the leak of cellular contents and the penetration of the SeNPs into the cell. Indeed, SEM assessment of the SeNP-treated bacteria detected significant damage on cell walls, such as denting, deformation, shrinkage, and cell disruption (Figure 5B–D,G,H). Furthermore, the cell width of *Enterobacter cloacae* (ATCC BAA3044), assessed by ImageJ, remarkably decreased when bacteria were treated with 30 µg/mL of SeNPs (Table 2), indicating leakage of cellular contents. Our results also revealed that a lower concentration of SeNPs was required to start causing damage in Gram-positive bacterium *Listeria monocytogens* (ATCC15313), and the damage was more severe, compared to that in Gram-negative bacterium *Enterobacter cloacae* (ATCC BAA3044) (Figure 5B–D,G,H), consistent with the observation of a stronger effect against *Listeria monocytogens* (ATCC15313).

In addition, nanoparticles have been shown to result in protein and DNA damage, increases in reactive oxidative species (ROS) level, and impairment of cellular metabolism and function once they enter bacteria [40]. There was no direct evidence proving the presence of SeNPs inside bacteria in our study; however, EDS analysis confirmed that SeNPs attached to the cell debris (Figure 5I,J). Thus, it is possible that SeNP penetration may also cause cell damage and cell death by disrupting biological molecules’ function and enhancing ROS production. The changes in cell morphology, together with the observation that cellular debris contained selenium, indicated that SeNPs may have disintegrated cell membranes and caused bacterial leakage, leading to cell shrinking, as well as eventual cell lysis and death (Figure 5).

Nanoparticle size and shape are important determinants of the functions of SeNPs, by affecting how efficiently the SeNPs bind to bacterial surfaces and are internalized [40,42,44,45]. The optimum size of nanoparticles for uptake into cells by endocytosis was shown to be around 50 nm with a spherical shape, but they could efficiently bind to cell membranes with sizes between 30 nm and 50 nm [44,45]. The SeNPs in our study had a spherical shape and an average diameter of 22.8 ± 4.7 nm (Figure 2), which is close to the lower borderline of the range. The relatively smaller size should still allow bacterial cells to internalize the SeNPs, though probably not at a maximum rate [44,45]. However, size and shape may not be a key factor that led to the different antibacterial action of SeNPs on Gram-positive and Gram-negative bacteria in the current study, since all bacteria were treated with the same SeNPs. Thus, the BSA coating may have played a critical role in mediating the different bactericidal effects of BSA-coated SeNPs on Gram-negative and Gram-positive bacteria.

Our finding that SeNPs exhibited a stronger inhibition on the growth of Gram-positive bacteria is not consistent with literature reports that SeNPs displayed strong antibacterial effects against both Gram-positive and -negative bacteria [2,4,6,10,29,54,55]. In contrast to these studies, we did not see an evident bactericidal activity against *Salmonella enterica* (ATCC49284), *Enterobacter cloacae* (ATCC BAA3044), or *Escherica coli* (ATCC BAA2326) (Table 1). Moreover, in the cases that there was an antibacterial effect in the present study, it was not as strong as those of biogenic SeNPs reported in the literature [2,10,54,55]. We were even unable to obtain the MIC and MBC of SeNPs against any of the test bacteria, due to the inability of SeNPs to completely kill the bacteria, but our results were consistent with reports by Chung, in which zeta potential, MIC, and MBC were not reported [30]. The differences in SeNP functionality may be partly attributed to the different methods of SeNP synthesis and differences in test bacteria strains used in these studies [2,4,6,10,54,55]. In the biogenic methods used in these studies [2,10,54,55], the reaction buffer contained different functional biomolecules, such as proteins, polysaccharides, and lipids, which capped the SeNPs with different charges than those of the BSA-coated SeNPs in our study. These coating molecules may exert a stronger affinity than BSA does with the same bacteria, thus enhancing the antibacterial activity of the coated SeNPs. It is interesting that SeNPs with either a positive [4] or negative zeta potential [2,54,55] displayed stronger antimicrobial activity than that of SeNPs in the present study, implying that regional interaction between SeNPs and biomolecules on bacterial cell walls or cell membranes probably played a more important role in attracting the SeNPs to the bacterial surfaces.

The size of SeNPs may also have an impact on their efficiency. The SeNPs in the current study, with an average diameter of 22.8 ± 4.7 nm, may not be internalized by bacteria as efficiently as those tested in the literature [2,4,30,54,55], since it was demonstrated that an optimum size for internalization was about 50 nm [44,45]. In the study led by Chung [30], SeNPs with a diameter around 40 nm showed a stronger effect than those with a smaller size. Filipovic reported that BSA-coated SeNPs with a diameter of 70–100 nm but a positive zeta potential [4] showed stronger antimicrobial activity than what we observed for the SeNPs in the present study. The SeNPs with larger sizes in these studies may be more likely to be internalized than the relatively smaller SeNPs in our study were. The size difference may partially account for the weaker function of SeNPs reported in the current study, compared to those reported in the literature [4,54,55].

The failure of SeNPs to fully kill *Listeria moncytogens* (ATCC15313), *Staphylococus aureus* (ATCC12600), *Staphylococus epidermidis* (ATCC 700583), *Vibrio alginolyticus* (ATCC 33787), and *Salmonella enterica* (ATCC19585) suggested that these bacteria can tolerate high concentration of SeNPs, even if SEM examination revealed that an increase in SeNP concentration did cause severe damage to bacteria (Figure 5D,G,H). The mechanism of this tolerance is not clear. It was suggested that *Vibrio parahaemolyticus* could develop capsules under unfavorable conditions [56]. Thus, it is possible that bacteria such as *Vibrio parahaemolyticus* might develop some type of protection against the damaging effect, which may allow them to escape the toxic effect of SeNPs at high concentrations. In addition, bacteria may recover from some damage, such as the increase in permeability, when they grew on solid growth medium in the absence of SeNPs. Whether the bacteria can survive longer durations of SeNP treatment remains to be tested. Furthermore, in the current study, as well as in many of the cited references [2,4,6,10,27,28,30,54,55], high concentrations of SeNPs were needed to inhibit or kill bacteria. Thus, attention should be directed to the toxicity of selenium nanoparticles if they are applied in food preservatives, since high doses of selenium led to toxicity in humans [57].

## 5. Conclusions

In summary, this is the first study examining the antibacterial activity of chemically synthesized SeNPs against as many as 10 different food-borne bacteria. BSA-coated SeNPs displayed inhibitory effects on the growth of some common food-borne pathogens, but they did not completely stop the growth of any of the test bacteria in the present study. The observed antibacterial activity may be associated with damage to cell walls and cell membranes, and the subsequent cell membrane leakage, as well as cell lysis. However, a single application of SeNPs synthesized through the reduction of SeO_3_^+2^ by vitamin C in the presence of BSA may not be strong enough to prevent bacteria-mediated food spoilage. A combination of SeNPs with other food preservatives may be necessary. Furthermore, size, shape, and surface chemical properties should be included in the criteria for selecting SeNPs as food additives to preserve food quality.

## Figures and Tables

**Table 1:** Antibacterial Activity of SeNPs against Bacteria.

Treatment	Kanamycin (µg/mL)	100	0	0	0	0	0	0	0	0
	SeNPs (µg/mL)	0	0	0.5	1	2.5	5	10	15	30
**CFU (×10^7^) after treatment**	*L. Monocytogens* (ATCC15313)	0	65.0 ± 13.1	54.5 ± 6.3 **	32.5 ± 8 ***	14.2 ± 4.7 ***	17.8 ± 16.6 ***	12.0 ± 8.0 ***	10.0 ± 3.6 ***	10.8 ± 6.0 ***
	*S. aureus* (ATCC12600)	0	68.0 ± 11.5	57.8 ± 13.4	74.4 ± 9.8	51.8 ± 11.3	63.0 ± 17.3	39.2 ± 5.7 *	33.2 ± 10.1 *	27.6 ± 16.7 ***
	*S. epidermidis* (ATCC 700583)	0	57.7 ± 9.3	41.9 ± 13.0	36.1 ± 7.7 **	26.9 ± 7.9 ***	24.1 ± 6.2 ***	26.6 ± 9.3 **	31.8 ± 9.2 **	31.1 ± 8.4 **
	*E. faecalis* (ATCC 19433)	0	45.1 ± 10.4	51.5 ± 19.9	50.9 ± 20.6	43.1 ± 16.6	38.0 ± 19.7	41.7 ± 10.6	41.5 ± 10.8	42.5 ± 12.8
	*V. alginolyticus* (ATCC 33787)	0	65.0 ± 13.9	57.5 ± 10.9	47.0 ± 11.9	36.5 ± 15.2 *	38.9 ± 10.7 **	33.8 ± 11.8 **	31.9 ± 13.2 **	31.2 ± 13.3 **
	*S. enterica* (ATCC19585)	0	56.3 ± 8.8	57.2 ± 5.9	64.0 ± 13.5	50.8 ± 11.1	52.3 ± 6.7	48.5 ± 10.4	39.0 ± 13.1 *	39.6 ± 8.1 *
	*V. Parahaemolyticus* (ATCC43996)	0	18.5 ± 7.5	21.2 ± 8.7	24.4 ± 13.8	17.7 ± 6.8	18.9 ± 6.6	16.1 ± 5.8	18.4 ± 5.5	24.5 ± 6.8
	*S. enterica* (ATCC49284)	0	33.1 ± 10.5	46.2 ± 7.0	39.2 ± 11.7	35.6 ± 18.7	35.6 ± 11.7	32.9 ± 4.4	28.1 ± 9.1	34.7 ± 12.5
	*E. cloacae* (ATCC BAA3044)	126.0 ± 21.2 ^#^	186.8 ± 50.8	142.1 ± 25.1	182.3 ± 19.1	175.2 ± 58.7	182.1 ± 55.0	181.9 ± 50.7	188.9 ± 29.7	202.6 ± 62.8
	*E. coli* (ATCC BAA2326)	0	196.1 ± 37.2	191.6 ± 59.6	218.9 ± 9.31	201.1 ± 39.7	173.5 ± 6.30	172.9 ± 54.3	210.9 ± 9.63	240.6 ± 92.2

#: Carbenicillin (100 µg/mL) and Kanamycin (100 µg/mL). CFU: colony-forming unit. *: *p* < 0.05, **: *p* < 0.01, ***: *p* < 0.001. The results were obtained from five different assays.

**Table 2:** Bacterial width after Treatment of SeNPs.

Bacterium	*L. Monocytogens* (ATCC15313)				*Enterobacter cloacae* (ATCC BAA3044)			
SeNP Concentration (µg/mL)	0	5	10	30	0	**5**	**10**	**30**
Width of bacteria (nm)	384.4 ± 45.0	391.0 ± 37.7	395.9 ± 46.1	378.8 ± 42.6	640.8 ± 47.6	661.8 ± 205.6	638.6 ± 74.2	594.9 ± 73.3 *
Number of bacteria (n)	64	66	66	43	54	45	44	65

*: *p* < 0.001, compared to width of bacteria without SeNP treatment.

## Data Availability

Data are available upon request.

## Supplementary Materials

The following supporting information can be downloaded at: https://www.mdpi.com/article/10.3390/microorganisms11061519/s1.

## References

[1] Skalickova S., Milosavljevic V., Cihalova K., Horky P., Richtera L., Adam V. (2017). Selenium nanoparticles as a nutritional supplement. Nutrition.

[2] Gunti L., Dass R.S., Kalagatur N.K. (2019). Phytofabrication of Selenium Nanoparticles from Emblica officinalis Fruit Extract and Exploring Its Biopotential Applications: Antioxidant, Antimicrobial, and Biocompatibility. Front. Microbiol..

[3] Wang M., Sun X., Wang Y., Deng X., Miao J., Zhao D., Sun K., Li M., Wang X., Sun W. (2022). Construction of Selenium Nanoparticle-Loaded Mesoporous Silica Nanoparticles with Potential Antioxidant and Antitumor Activities as a Selenium Supplement. ACS Omega.

[4] Filipovic N., Ušjak D., Milenković M.T., Zheng K., Liverani L., Boccaccini A.R., Stevanovic M.M. (2021). Comparative Study of the Antimicrobial Activity of Selenium Nanoparticles with Different Surface Chemistry and Structure. Front. Bioeng. Biotechnol..

[5] Lin W., Zhang J., Xu J.-F., Pi J. (2021). The Advancing of Selenium Nanoparticles Against Infectious Diseases. Front. Pharmacol..

[6] Sentkowska A., Pyrzyńska K. (2022). The Influence of Synthesis Conditions on the Antioxidant Activity of Selenium Nanoparticles. Molecules.

[7] Kieliszek M., Bano I., Zare H. (2022). A Comprehensive Review on Selenium and Its Effects on Human Health and Distribution in Middle Eastern Countries. Biol. Trace Elem. Res..

[8] Yang J., Wang J., Yang K., Liu M., Qi Y., Zhang T., Fan M., Wei X. (2018). Antibacterial activity of selenium-enriched lactic acid bacteria against common food-borne pathogens in vitro. J. Dairy Sci..

[9] Mates I., Antoniac I., Laslo V., Vicas S., Brocks M., Fritea L., Milea C., Mohan A., Cavalu S. (2019). Selenium nanoparticles: Production, characterization and possible applications in biomedicine and food science. UPB Sci. Bull. Ser. B Chem. Mater. Sci..

[10] Salem S.S. (2022). Bio-fabrication of Selenium Nanoparticles Using Baker’s Yeast Extract and Its Antimicrobial Efficacy on Food Borne Pathogens. Appl. Biochem. Biotechnol..

[11] Gonca S. (2013). Selenium: A Micronutrient Essential for Maintaining Human Health. Med. Sci..

[12] Mehdi Y., Hornick J.-L., Istasse L., Dufrasne I. (2013). Selenium in the Environment, Metabolism and Involvement in Body Functions. Molecules.

[13] Kieliszek M. (2019). Selenium–Fascinating Microelement, Properties and Sources in Food. Molecules.

[14] Burk R.F., Hill K.E. (2009). Selenoprotein P– Expression, Functions, and Roles in Mammals. Biochim. Biophys. Acta.

[15] Labunskyy V.M., Hatfield D.L., Gladyshev V.N. (2014). Selenoproteins: Molecular Pathways and Physiological Roles. Physiol. Rev..

[16] Weekley C.M., Harris H.H. (2013). Which form is that? The importance of selenium speciation and metabolism in the prevention and treatment of disease. Chem. Soc. Rev..

[17] Hatfield D.L., Yoo M.H., Carlson B.A., Gladyshev V.N. (2009). Selenoproteins that function in cancer prevention and promotion. Biochim. Biophys. Acta Gen. Subj..

[18] Rayman M.P. (2000). The importance of selenium to human health. Lancet.

[19] Larsen P.R., Zavacki A.M. (2012). Role of the Iodothyronine Deiodinases in the Physiology and Pathophysiology of Thyroid Hormone Action, Translational Thyroidology/Review. Eur. Thyroid J..

[20] Xia Y., Tang G., Wang C., Zhong J., Chen Y., Hua L., Li Y., Liu H., Zhu B. (2020). Functionalized selenium nanoparticles for targeted siRNA delivery silence Derlin1 and promote antitumor efficacy against cervical cancer. Drug Deliv..

[21] Varlamova E.G., Turovsky E.A., Blinova E.V. (2021). Therapeutic Potential and Main Methods of Obtaining Selenium Nanoparticles. Int. J. Mol. Sci..

[22] Zhang J., Wang X., Xu T. (2008). Elemental Selenium at Nano Size (Nano-Se) as a Potential Chemopreventive Agent with Reduced Risk of Selenium Toxicity: Comparison with Se-Methylselenocysteine in Mice. Toxicol. Sci..

[23] Tan T.L.C., Hinchman A., Williams R., Tran P.A., Fox K. (2018). Nanostructured biomedical selenium at the biological interface (Review). Biointerphases.

[24] Estevez H., Garcia-Lidon J.C., Luque-Garcia J.L., Camara C. (2014). Effects of chitosan-stabilized selenium nanoparticles on cell proliferation, apoptosis and cell cycle pattern in HepG2 cells: Comparison with other selenospecies. Colloids Surf. B Biointerfaces.

[25] Hosnedlova B., Kepinska M., Skalickova S., Fernandez C., Ruttkay-Nedecky B., Peng Q., Baron M., Melcova M., Opatrilova R., Zidkova J. (2018). Nano-selenium and its nanomedicine applications: A critical review. Int. J. Nanomed..

[26] Rajkumar K., Mvs S., Koganti S., Burgula S. (2020). Selenium Nanoparticles Synthesized Using Pseudomonas stutzeri (MH191156) Show Antiproliferative and Anti-angiogenic Activity Against Cervical Cancer Cells. Int. J. Nanomed..

[27] El-Deeba B., Al-Talhib A., Mostafac N., Abou-assyd R. (2018). Biological Synthesis and Structural Characterization of Selenium Nanoparticles and Assessment of Their Antimicrobial Properties. Am. Sci. Res. J. Eng. Technol. Sci. (ASRJETS).

[28] Mosallam F.M., El-Sayyad G.S., Fathy R.M., El-Batal A.I. (2018). Biomolecules-mediated synthesis of selenium nanoparticles using *Aspergillus oryzae* fermented Lupin extract and gamma radiation for hindering the growth of some multidrug-resistant bacteria and pathogenic fungi. Microb. Pathog..

[29] Alghuthaymi M.A., Diab A.M., Elzahy A.F., Mazrou K.E., Tayel A.A., Moussa S.H. (2021). Green Biosynthesized Selenium Nanoparticles by Cinnamon Extract and Their Antimicrobial Activity and Application as Edible Coatings with Nano-Chitosan. J. Food Qual..

[30] Chung S., Zhou R., Webster T.J. (2020). Green Synthesized BSA-Coated Selenium Nanoparticles Inhibit Bacterial Growth While Promoting Mammalian Cell Growth. Int. J. Nanomed..

[31] Fouda A., Hassan S.E.D., Eid A.M., Abdel-Rahman M.A., Hamza M.F. (2022). Light enhanced the antimicrobial, anticancer, and catalytic activities of selenium nanoparticles fabricated by endophytic fungal strain, Penicillium crustosum EP-1. Sci. Rep..

[32] Kamble S., Agrawal S., Cherumukkil S., Sharma V., Jasra R.V., Munshi P. (2022). Revisiting Zeta Potential, the Key Feature of Interfacial Phenomena, with Applications and Recent Advancements. ChemistrySelect.

[33] Liu J. (2005). Scanning transmission electron microscopy and its application to the study of nanoparticles and nanoparticle systems. Microscopy.

[34] Bhattacharya R., Saha S., Kostina O., Muravnik L., Mitra A. (2020). Replacing critical point drying with a low-cost chemical drying provides comparable surface image quality of glandular trichomes from leaves of *Millingtonia hortensis* L. f. in scanning electron micrograph. Appl. Microsc..

[35] Alagesan V., Venugopal S. (2019). Green Synthesis of SeNPs Using Leaves Extract of Withania somnifera and Its Biological Applications and Photocatalytic Activities. BioNanoSci.

[36] Sheeana G., Dragana S., Robert J.H., Moore R.J., Chapman J. (2017). The synthesis and characterization of highly stable and reproducible selenium nanoparticles. Inorg. Nano-Met. Chem..

[37] Mu X., Yan C., Tian Q.W., Lin J., Yang S. (2017). BSA-assisted synthesis of ultrasmall gallic acid–Fe (III) coordination polymer nanoparticles for cancer theranostics. Int. J. Nanomed..

[38] Bhattacharjee S. (2016). DLS and zeta potential—What they are and what they are not?. J. Control. Release.

[39] Toubhans B., Gazze S.A., Bissardon C., Bohic S., Gourlan A.T., Gonzalez D., Charlet L., Conlan R.S., Francis L.W. (2020). Selenium nanoparticles trigger alterations in ovarian cancer cell biomechanics. Nanomed. Nanotechnol. Biol. Med..

[40] Escobar-Ramírez M.C., Castañeda-Ovando A., Pérez-Escalante E., Rodríguez-Serrano G.M., Ramírez-Moreno E., Quintero-Lira A., Contreras-López E., Añorve-Morga J., Jaimez-Ordaz J., González-Olivares L.G. (2021). Antimicrobial Activity of Se-Nanoparticles from Bacterial Biotransformation. Fermentation.

[41] Yuan Q., Bomma M., Hill H., Xiao Z. (2022). Expression of Rhizobium tropici phytochelatin synthase in Escherichia coli resulted in increased bacterial selenium nanoparticle synthesis. J. Nanopart. Res..

[42] Sentkowska A., Pyrzyńska K. (2023). Antioxidant Properties of Selenium Nanoparticles Synthesized Using Tea and Herb Water Extracts. Appl. Sci..

[43] Toprakcioglu Z., Wiita E.G., Jayaram A.K., Gregory R.C., Knowles T.P.J. (2023). Selenium Silk Nanostructured Films with Antifungal and Antibacterial Activity. ACS Appl. Mater. Interfaces.

[44] Varlamova E.G., Gudkov S.V., Plotnikov E.Y., Turovsky E.A. (2022). Size-Dependent Cytoprotective Effects of Selenium Nanoparticles during Oxygen-Glucose Deprivation in Brain Cortical Cells. Int. J. Mol. Sci..

[45] Foroozandeh P., Aziz A.A. (2018). Insight into Cellular Uptake and Intracellular Trafficking of Nanoparticles. Nanoscale Res. Lett..

[46] Touliabah H.E., El-Sheekh M.M., Makhlof M.E.M. (2022). Evaluation of Polycladia myrica mediated selenium nanoparticles (PoSeNPS) cytotoxicity against PC-3 cells and antiviral activity against HAV HM175 (Hepatitis A), HSV-2 (Herpes simplex II), and Adenovirus strain 2. Front. Mar. Sci..

[47] Silhavy T.J., Kahne D., Walker S. (2010). The bacterial cell envelope. Cold Spring Harb. Perspect. Biol..

[48] Slavin Y.N., Asnis J., Häfeli U.O., Bach H. (2017). Metal nanoparticles: Understanding the mechanisms behind antibacterial activity. J. Nanobiotechnol..

[49] Chung Y.C., Su Y.P., Chen C.C., Jia G., Wang H.L., Wu J.C.G., Lin J.G. (2004). Relationship between antibacterial activity of chitosan and surface characteristics of cell wall. Acta Pharmacol. Sin..

[50] Gottenbos B., Grijpma D.W., Van Der Mei H.C., Feijen J., Busscher H.J. (2001). Antimicrobial effects of positively charged surfaces on adhering Gram-positive and Gram-negative bacteria. J. Antimicrob. Chemother..

[51] Dziarski R. (1994). Cell-bound albumin is the 70-kDa peptidoglycan-, lipopolysaccharide-, and lipoteichoic acid-binding protein on lymphocytes and macrophages. J. Biol. Chem..

[52] Prasad A.R., Basheer S.M., Gupta I.R., Elyas K.K., Joseph A. (2020). Investigation on Bovine Serum Albumin (BSA) binding efficiency and antibacterial activity of ZnO nanoparticles. Mater. Chem. Phys..

[53] Espinosa-Cristóbal L.F., Martínez-Castañón G.A., Loyola-Rodríguez J.P., Niño-Martínez N., Ruiz F., Zavala-Alonso N.V., Lara R.H., Reyes-López S.Y. (2016). Bovine serum albumin and chitosan coated silver nanoparticles and its antimicrobial activity against oral and nonoral bacteria. J. Nanomater..

[54] Rao C.K., Mangamuri U.K., Sikharam A.S., Devaraj K., Kalagatur N.K., Kadirvelu K. (2022). Biosynthesis of Selenium Nanoparticles from Annona muricata Fruit Aqueous Extract and Investigation of their Antioxidant and Antimicrobial potentials. Curr. Trends Biotechnol. Pharm..

[55] El-Zayat M.M., Eraqi M.M., Alrefai H., El-Khateeb A.Y., Ibrahim M.A., Aljohani H.M., Aljohani M.M., Elshaer M.M. (2021). The Antimicrobial, Antioxidant, and Anticancer Activity of Greenly Synthesized Selenium and Zinc Composite Nanoparticles Using Ephedra aphylla Extract. Biomolecules.

[56] Bian S., Zeng W., Li Q., Li Y., Wong N.K., Jiang M., Zuo L., Hu Q., Li L. (2021). Genetic Structure, Function, and Evolution of Capsule Biosynthesis Loci in *Vibrio parahaemolyticus*. Front. Microbiol..

[57] MacFarquhar J.K., Broussard D.L., Melstrom P., Hutchinson R., Wolkin A., Martin C., Burk R.F., Dunn J.R., Green A.L., Hammond R. (2010). Acute selenium toxicity associated with a dietary supplement. Arch. Intern. Med..

